# Preservation of Animal Food

**Published:** 1862-10

**Authors:** Samuel Ainsworth

**Affiliations:** Villa Mont Blanc, Spring-grove


### PRESERVATION OF ANIMAL FOOD.

To the Editor of the Lancet,

SIR:—Knowing the avidity with which you seek information upon subjects having for their object the general good, and more especially if that subject is dependent upon chemical scientific researches, I am induced to go out of my usual course by forwarding this letter on the important subject of the preservation of animal food in its uncooked state.

I was invited a few days since, by Mr. Richard Jones, of Botolph-lane, City, to inspect his specimens of uncooked preserved joints of meat. A simple statement of facts will explain more fully the nature of this application of science to the requirements of man than any description of mine will convey. I was shown several hundred tins containing hams and bacon, hermetically sealed, from which the whole of the air had been extracted, and the vessel filled with a non-decomposing element, to be shipped for consumption to India, China, and other tropical climates. It will, doubtless, be said that nothing is more simple than to preserve that which has been already cured by salting; at all events, the success attending this branch of the business paved the way for more astonishing results, for in consequence of the perfectly-preserved state of this mildly-cured animal substances, it was determined to try the same means upon fresh *uncooked* meat. The various specimens inspected are a proof of the superiority of this method over every other that has been hitherto brought under the notice of the public.

It appears that Mr. Jones has been engaged on his investigation into this subject for several years. He, from the onset, felt satisfied that if means could be adopted by which every pound of meat should have its given quantity of preservative gas, and the vessel containing the meat could be deprived of the whole of its oxygen, the result must be an entire absence of decomposition. Such a theory put into practice is entirely confirmed by results. As a proof, there are a large number of tins containing beef, legs of mutton, fowls, sausages, fish, &c., all of which have been there for months, put to every test of temperature, yet remain perfectly free from decomposition. It was found by experience that tin, although the most convenient vessel, had its objection, and it was determined to place the material to be acted upon in earthenware pans or jars. The result was most satisfactory; the meat could not be distinguished, either before or after cooking, from that just obtained from the butcher's, although encased for several months previously. Such was the success attending the placing it in earthenware that Mr. Jones determined to expose the meat thus preserved under glass shades for the Exhibition. For this purpose he merely secured a fresh leg of mutton to a porcelain fish strainer, and fastened this to a small board, into which were screwed some tin stands; these were soldered to a tin-plate bottom, which was again soldered to a rim of tin, which had been previously secured to the bottom of the glass shade by means of hot-cement. The air was then withdrawn from the inside of the shade, a non-decomposing gas introduced, and the aperture sealed up. The apparatus arranged to carry out this purpose protects the fragile glass, whatever may be the amount of atmospheric pressure. During this operation the glass becomes dim in consequence of the deposit of vapor from the meat upon the inside of the glass; yet the contents are distinctly visible, neither increasing, diminishing, or changing their natural appearance.

These are now in the eastern annex of the International Exhibition. Several of these glass shades, containing beef, mutton, fowls, pigeons, salmon, soles, &c., are well worthy the inspection of our naval, military, and hospital surgeons. Who knows but that meat now allowed to rot in the Brazils and other distant countries for want of means of preserving it, may be brought to this country, and be a cheap food for our over-crowded population! There is a large field open to inquiry, and we have only now arrived at the beginning of large results. From looking at the specimens of animal food, I am led to think that similar means applied to the preservation of pathological and anatomical specimens would add much to the beauty of our museums.

I am, Sir, yours respectfully,

SAMUEL AINSWORTH, F.L.S., &c.

Villa Mont Blanc, Spring-grove, Sept., 1862.

—*London Lancet.*

